# Combining Cell Technologies With Biomimetic Tissue Engineering Applications: A New Paradigm for Translational Cardiovascular Therapies

**DOI:** 10.1093/stcltm/szad002

**Published:** 2023-02-20

**Authors:** Sarah E Motta, Marcy Martin, Eric K N Gähwiler, Valery L Visser, Polina Zaytseva, Arian Ehterami, Simon P Hoerstrup, Maximilian Y Emmert

**Affiliations:** Institute for Regenerative Medicine (IREM), University of Zurich, Zurich, Switzerland; Institute for Regenerative Medicine (IREM), University of Zurich, Zurich, Switzerland; Institute for Regenerative Medicine (IREM), University of Zurich, Zurich, Switzerland; Institute for Regenerative Medicine (IREM), University of Zurich, Zurich, Switzerland; Institute for Regenerative Medicine (IREM), University of Zurich, Zurich, Switzerland; Institute for Regenerative Medicine (IREM), University of Zurich, Zurich, Switzerland; Institute for Regenerative Medicine (IREM), University of Zurich, Zurich, Switzerland; Wyss Zurich, University and ETH Zurich, Zurich, Switzerland; Institute for Regenerative Medicine (IREM), University of Zurich, Zurich, Switzerland; Wyss Zurich, University and ETH Zurich, Zurich, Switzerland; Charité Universitätsmedizin Berlin, Berlin, Germany; Deutsches Herzzentrum der Charité (DHZC), Dept of Cardiothoracic and Vascular Surgery, Berlin, Germany

**Keywords:** tissue engineering, human pluripotent stem cells, computational modeling, additive manufacturing

## Abstract

Cardiovascular disease is a major cause of morbidity and mortality worldwide and, to date, the clinically available prostheses still present several limitations. The design of next-generation regenerative replacements either based on cellular or extracellular matrix technologies can address these shortcomings. Therefore, tissue engineered constructs could potentially become a promising alterative to the current therapeutic options for patients with cardiovascular diseases. In this review, we selectively present an overview of the current tissue engineering tools such as induced pluripotent stem cells, biomimetic materials, computational modeling, and additive manufacturing technologies, with a focus on their application to translational cardiovascular therapies. We discuss how these advanced technologies can help the development of biomimetic tissue engineered constructs and we finally summarize the latest clinical evidence for their use, and their potential therapeutic outcome.

## Graphical Abstract

**Graphical Abstract:**
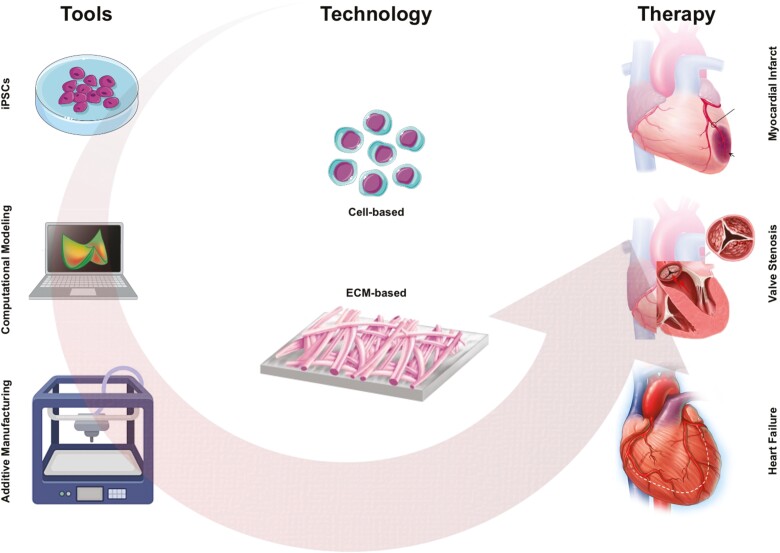

**Significance Statement**

Tissue-engineered constructs may revolutionize our approach to clinical practice and set a new boundary for the treatment of cardiovascular diseases. The combination of cutting-edge technologies together with cell-based and ECM-based therapies will ultimately provide regenerative, self-repairing, and growing prostheses; a potential solution to the increasing needs of patients, and particularly children. As recently demonstrated in preclinical and early clinical studies, pre-seeded and decellularized tissue-engineered replacements may overcome the current clinical shortcomings, by providing biomimetic substitutes that can completely integrate within the host and the native tissues, grow, and remodel over time.

## Introduction

Cardiovascular disease comprises the major spectrum of worldwide deaths every year.^1^ Many of these progressive diseases lead to the gradual deterioration of the affected tissues and, at their end-stage, will need to be replaced. Despite the extensive technological and pharmacological advancements to date, clinically-available prostheses (eg, autografts, allografts, xenografts, or synthetic constructs) still suffer from several inherent limitations such as donor shortage, lifelong anticoagulation treatment, immune rejection, and limited durability.^2^ Cell-based as well as extracellular matrix (ECM)-based therapies represent a potential game-changing approach to promote cardiac regeneration following heart valve damage, congenital heart diseases, myocardial infarction, or heart failure.^3^ Over the past decades, advanced tissue engineering tools such as human induced pluripotent stem cells (hiPSCs),^4^ adaptive biomaterials,^5^ computational modeling,^6,7^ and 3D printing technologies^8^ granted the further development of regenerative therapies that contributes to self-repairing prostheses. The ultimate goal of these biomimetic approaches is to regenerate cardiovascular tissues by restoring or improving the original tissue function. Current tissue engineering strategies involve the use of natural or synthetic matrices, containing either primary donor cells or a decellularized scaffold material, both of which provide a porous polymeric environment that sustains cell adhesion, growth, and migration.^9^ However, such approaches have major limitations and carry disadvantages that may result in non-homogeneous and non-reproducible outcomes. A more mechanistic approach to understand the underlying phenomena of in vivo tissue remodeling and growth could endeavor functional regeneration and preserved long-term functionality of such prostheses.^10^ Computational modeling is therefore an indispensable tool to predict and guide the complex interplay between cells, tissue, and the cardiovascular environment. Indeed, recent studies have demonstrated that the integration of computational modeling into the experimental pipeline improves and supports a superior preclinical outcome of the designed matrices.^10,11^ Furthermore, the advent of additive manufacturing 3D bioprinting technology has made strides in advancing the field by allowing stem cells, biomimetic materials, and computational modeling tools to generate precisely organized geometries, which mimic their native counterparts and thereby potentially provide personalized prostheses.^8^ In this review, we aim to selectively highlight some of the most advanced technologies at our disposal that can be adopted to further improve and fasten the clinical translation of cardiac regeneration.

## Human iPSCs and 3D Microtissues for Cardiovascular Tissue Engineering

As highlighted in 2000 by Langer and Vacanti, classic cardiovascular tissue engineering approaches utilize primary cells directly isolated from donors to create regenerative ECM in vitro.^9^ The use of allogeneic cell sources might however cause a higher risk of non-homogeneous ECM deposition and composition, depending on the comorbidities of the patient and the performance of the isolated donor cells.^12^ The key disadvantage when using human primary cells is undeniably donor-to-donor variability, which can negatively affect cell growth, ECM stability, and immunocompatibility within the host. Furthermore, primary cells have a defined shelf-life and cannot be infinitely sub-cultured. To overcome these issues, human iPSCs (hiPSCs) have been introduced as an alternative cell source. The beauty of hiPSCs is their ability to be reprogrammed from individual patients, thus retaining patient-specific properties that circumvent the immunogenic response after their transplantation.^13,14^ Furthermore, hiPSCs have theoretically unlimited growth potential, which can help overcome protocols limitations in scaling up for clinical translation.

**Figure 1.**
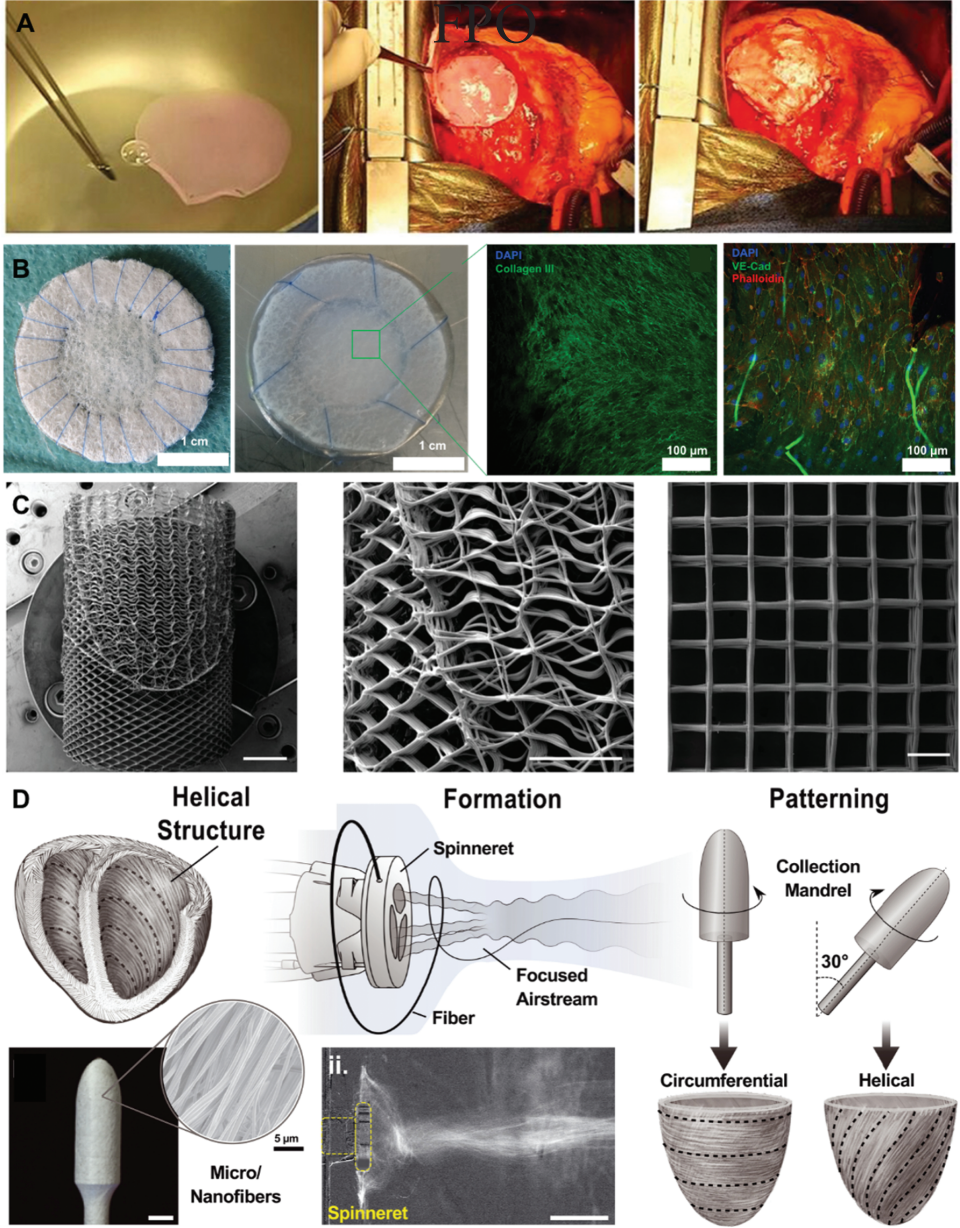
Examples of the most recent tissue engineering technologies. **A**: Representative figures of the implantation of a cardiovascular progenitor-loaded fibrin patch onto the epicardium of the infarcted area.^15^**B**: Macroscopical and microscopic appearance of hTEMs before and after in vitro culture.^16^**C**: Design and fabrication of tubular, spatially heterogeneous, PCL MEW scaffolds (scale bar: 5 mm).^17^**D**: Focused rotary jet spinning for producing helical structures.^18^ Figures were adapted and reprinted under Creative Commons CC-BY License and with permissions from the journals.

Clinical translation is rapidly evolving for hiPSCs-based cardiovascular medicine as researchers are currently harnessing the power of hiPSC-derived cardiomyocytes (iCMs) in finding therapies to improve cardio-protection or cardiac regeneration following tissue injury. Current clinical trials include the phase 1 ESCORT trial (NCT02057900), where fibrin gel loaded with human embryonic stem cells (ESC)-derived CD15^+^ lsl-1^+^ cardiovascular progenitors were transplanted onto the epicardium of a severely infarcted area, which resulted in sustained contractility improvement (Table 1 and Fig. 1A).^15^ Notably, none of the six patients showed arrhythmia, tumor formation, and nor immunoreaction.^15^ Current ongoing iCM-based clinical trials, as extensively reviewed elsewhere,^19^ include the BioVAT-HF (NCT04396899) in Germany and the HEAL-CHF (NCT03763136) in China, which aim to treat patients suffering from heart failure and are actively recruiting patients for either the surgical implantation of an iCM-based cardiac patch or the direct injection of iCMs to the epicardium, respectively (Table 1). The results of these trials are eagerly awaited. Additional clinical studies investigating new cell therapies approach to improve survival and cardiac function in patients with chronic left ventricular dysfunction secondary to myocardial infarction (HECTOR, NCT05068674) or congestive heart failure (NCT04982081) via endocardial injection is currently ongoing. On the other hand, application of iPCS-derived CM in form of spheroids or sheet is currently being investigated as treatment option for heart failure (LAPiS, NCT04945018) or ischemic cardiomyopathy (NCT04696328), respectively (Table 1).

**Table 1.** Clinical applications of pluripotent stem cell-derived CMs as a therapy.

Trial (year and number)	Phase	Cell therapy	Administration route	Disease	Main findings	Ref.
ESCORT (2018, NCT02057900)	1	Transplantation of human embryonic stem cell-derived CD15^+^ Isl-1^+^ progenitors	Epicardially delivered during surgical coronary revascularization	Severe heart failure	Sustained contractility, no arrhythmia, tumor formation, nor immunoreaction	15
BioVAT-HF (2020, NCT04396899)	2	iPSC-derived engineered human myocardium	Left-lateral mini-thoracotomy or other interventions	Ventricular assist tissue in terminal heart failure	N.A.	-
HEAL-CHF (2021, NCT03763136)	2	Allogeneic human pluripotent stem cell-derived CM	Epicardially injected during coronary artery bypass grafting	Severe chronic heart failure	N.A.	-
Treating congestive heart failure patients with human iPSC-derived CM through catheter-based endocardial injection (2021, NCT04982081)	1	Human iPSC-CM	Transcatheter endocardial injection system	Congestive heart failure	N.A.	-
HECTOR (2021, NCT05068674)	1	Human embryonic stem cell-derived CM	Transcatheter endocardial injection	Chronic ischemic left ventricular dysfunction	N.A.	-
LAPiS (2021, NCT04945018)	1/2	Human iPSC-CM spheroids	N.A.	Severe heart failure secondary to ischaemic heart disease	N.A.	-
Clinical trial of human (allogeneic) iPSC-derived CM sheet for ischemic cardiomyopathy (2021, NCT04696328)	1	iPSC-CM sheet	Transplantation	Ischemic cardiomyopathy	N.A.	-

Nonetheless, therapies involving the implantation or injection of iCMs come with limitations of their own. For example, the maturity of iCMs has remained in question as they are characteristically immature or fetal-like in both morphology and electrophysiology. Currently, there are methods to further mature iCMs by using in vitro stimuli such as mechanical or electrical training, or increased culture periods.^20^ Another bottleneck that influences these approaches is the allogenic immune rejection by the recipient, as autologous transplantation is still costly and time-consuming. Therefore, banking for human leukocyte antigen (HLA) matching has been established but needs to be further evaluated. Developing less immunogenic hiPSC lines represents an alternative strategy, for instance, HLA class I and II genes are knocked out while HLA-E is overexpressed.^21^ The successful development of safe and effective immunocompatible strategies are essential to facilitate the clinical development of implantation hiPSC-based therapy. Furthermore, the minimal preservation of transplanted cells in dysfunctional or ischemic tissue currently limits the clinical effectiveness of iCM-based therapies. iCMs that are transplanted or injected either fail to reach the target tissue or perish during or shortly after use. Intriguingly, co-transplantation of non-myocyte and iCMs. In a rat myocardial infarction model has resulted in recent advancements.^22^ Compared to iCMs alone, co-transplantation improved graft size, vascular density, and maturation.^22^

Similar to co-transplantation efforts, cardiovascular 3D microtissues that contain hiPSC-derived cardiomyocytes, endothelial cells, and/or cardiac fibroblasts are emerging in fields such as drug discovery, toxicity testing, and precision medicine.^23^ Furthermore, these microtissues represent an exciting alternative to animal research.^24-26^ Specifically, microtissues are able to provide a microenvironment, including multi-lineage cells, ECM, and secreted paracrine factors, specific for each organ or tissue type.^27^ Cardiac microtissues have been found to have better tissue patterning, biological complexity, as well as a more mature cardiomyocyte phenotype when compared to their 2D counterparts.^28,29^ Studies demonstrating the direct comparison between 2D iCM and 3D cardiac microtissues have shown critical differences in models of genetic disorders such as hypertrophic cardiomyopathy (HCM) and dilated cardiomyopathy (DCM). The myosin heavy chain (MYH7) E848G mutation in familial HCM is an example of the usefulness of 3D cultures. Despite the E848G mutation not exhibiting contractile dysfunction in iCMs, their pathological phenotype was obvious in 3D microtissues.^30^ Similar research revealed that iCM contractile assays for titin-truncating mutations, which are frequent in genetic DCM onset, showed no abnormality.^31^ However, mutant 3D cardiac microtissue contraction had only half the strength as healthy controls.^31^ Despite the continuous research efforts, 3D microtissues are still limited in clinical translation. Even with our current advancements, microtissues do not accurately represent the physiology of adult organs as they do not contain the cardiac conduction system.^32^ Furthermore, diffusion or active perfusion remains insufficient, thereby reducing the turnover of cell debris and leading to the formation of a necrotic core if grown too large. Finally, there is the question of cell retention as well as tumorigenicity of hiPSC-derived microtissues when implanted into patients. An alternative solution could be the use of cell-free constructs, by decellularization of the microtissues before implantation to use as off-the-shelf available scaffolds. Similarly, secreted factors harvested from hiPSCs and microtissues have demonstrated potential for therapeutic use.^33^ Overall, characterization standards, which include specific protocols for stem cell differentiation, microtissue components, and GMP-compliant regulatory measures for safety and efficacy must be clearly defined before clinical translation of these approaches can move forward.^12^

## Decellularized Tissue Engineered Matrices

In parallel to hiPSC-based approaches, ECM-based technologies are also gaining attention for the design of suitable patient-specific adaptive biomaterials. Particularly, human cell-derived tissue-engineered matrices (hTEMs) being free of xenogeneic antigens could represent a promising candidate and holds great clinical translation potential^16,34-36^ (Fig. 1B). By looking closely at the field of heart valve prostheses, the first tissue-engineered decellularized human heart valve was introduced in Europe as an alternative to the clinically available biological (xenogeneic) prostheses.^34^ The in vivo application of decellularized pulmonary and aortic valve homografts was successful in both preclinical and clinical studies, showing excellent performance and spontaneous recellularization^37-40^ (ARISE: NCT02527629 and ESPOIR: NCT02035540, Table 2). Despite the great promise of human heart valve homografts, the issue of donor shortage still limits the broader clinical application of these prostheses and results in the need for alternative sources.^34^ To this end, decellularized xenografts generated from porcine small intestinal submucosa (CorMatrix) or porcine decellularized heart valves (Matrix P, Synergraft) have been used in preclinical and clinical studies^34^ (Table 2). However, the xenografts demonstrated inconsistent findings that range from promising performance and recellularization to inflammation, endocarditis, and death. The reasons underlying the poor performance or failure of xenografts are multifactorial and can be connected to host-specific factors (eg, health status, age, etc.), host-specific anatomy (eg, hemodynamics, annulus size, etc.), or importantly, to tissue-specific factors (eg, tissues from animal origin, presence of antigenic debris, incomplete decellularization, etc.).^34^ Remnants of cellular debris, traces of DNA, and the presence of xenogeneic collagen in the matrix are sufficient to elicit an immune response in the host that could lead to the final failure of the prosthesis.^44^ Calcification is one of the major drawbacks of the currently available biological prostheses resulting from this inflammatory reaction. Glutaraldehyde fixation is used to reduce the antigenicity of xenografts. However, this causes the valves to gradually degenerate and would need a replacement within 10–20 years, an undesirable option, especially for pediatric patients.^45^ Thus, the aim to design acellular xenogeneic-free cardiovascular replacements with self-renewal and regenerative properties may overcome significant clinical challenges.^46^

**Table 2.** Clinical applications of tissue-engineered technologies.

Trial or graft (year)	Prosthesis	Procedure	Mean age (years)	Main findings	Ref.
Decellularized homograft-based valve replacements					
ARISE (2020, NCT02527629)	Decellularized aortic homograft	SAVR	33.6 ± 20.8	Decellularized aortic homografts are safe for aortic valve replacement with excellent hemodynamics in the short follow-up period.	37,39
ESPOIR (2022, NCT02035540)	Decellularized pulmonary homograft	SPVR	Range 2-38	5-year data show excellent performance for decellularized pulmonary homografts with low rates of adverse events.	40
Decellularized xenograft-based valve replacements					
Porcine SIS (2020, NCT02397668)	Tricuspid valve	Surgical tricuspid replacement	NA	Good valve performance, mild regurgitation.	34
Porcine decellularized pulmonary valve (2019)	Decellularized pulmonary valve	SPVR	57 ± 11	Valvular dysfunction and ventricular failure.	34
Decellularized hTEM-based vascular grafts					
Humacyte (2022, NCT01744418)	Bioengineered human acellular vessel	Hemodialysis	18-80	Phase II long-term follow-up study shows that the human acellular vessel provides durable and functional hemodialysis access for patients with end stage renal disease.	41
Bioresorbable polymer-based vascular grafts and valve replacement					
Tissue-engineered vascular graft (2020, IDE14127)	Pre-seeded biodegradable conduit	Fontan conduit	N.A.	Promising function and potential for growth, remodeling, and repair. Longer follow-up studies are necessary.	42
Xeltis vascular graft (2017, NCT02377674)	Biodegradable conduit, COR-VG-001	Fontan conduit	4-12	No device-related adverse events were reported. Anatomical and functional stability in all patients at 12 months.	43
Xplore-1 (2020, NCT02700100)	Biodegradable polymeric valve	SPVR	2-12	No major clinical events and no signs of aneurysm or stenosis at 2 years follow-up. Moderate or severe regurgitation detected.	34
Xplore-2 (2020, NCT03022708)	Biodegradable polymeric valve	SPVR	2-9	Valve stenosis was reported at 1-year follow-up.	^34^

The use of human primary cells or hiPSCs within cardiac tissue-engineered constructs allows for a xenogeneic-free and patient-specific approach that significantly reduces the chances of implant rejection due to a foreign body response.^12,47,48^ Stem cells derived from various tissues of the patient, for example, skin, dental tissue, peripheral blood, and urine can be used for this purpose. Moreover, practical and reliable methods for handling hiPSCs under xenogeneic-free culture conditions need to be standardized to facilitate clinical translation.

## Computational Modeling Strategies for Precision Cardiovascular Tissue Engineering

The integration of computational modeling in stem cell-based tissue engineering therapies could dramatically increase our understanding into the causes of donor-to-donor variability and tissue remodeling outcomes, thereby helping the prediction of the in vivo evolution and providing superior preclinical and clinical performance of the resulting tissue-engineered prostheses.^49^ By continuously adapting to the received mechanical stimuli, cells are known to be the drivers of tissue growth and remodeling mechanisms.^10^ Evidence has shown that computational modeling at the cellular level has the opportunity to include factors mimicking cell–cell, cell–matrix, or cell–biomaterial interplay to explain in vivo phenomena. This increased understanding can be used to harness the cellular mechanisms responsible for cell behavior variability and their downstream effects on the ECM. In the context of cardiac tissue engineering, computational modeling of the electrophysiological behavior of hiPSCs-derived cardiomyocytes revealed that such cells are able to modulate the electrical conduction system in tissue-engineered constructs, thereby affecting the coordination of tissue remodeling.^50^ In other words, the successful integration of biochemical-, mechanical-, and physical stimuli in the mechanistic computational models can lead to the understanding of the phenotypic switches of hiPSC-derived cardiac myocytes, fibroblasts, or smooth muscle cells that finally affect the remodeling of tissue-engineered constructs.^51-53^ This multiscale modeling can predict the mechanical behavior at the tissue-level of the tissue-engineered construct, thus unraveling important parameters to prevent wear and tear of heart valves or other functional damages.^7,11,54,55^ Furthermore, these modeling tools can be used to study the effect of a hydrogel-based tissue-engineered construct cultured with hiPSC-derived cells providing information on the contractility and mechanical behavior of cardiac tissue, which is considered a potential treatment option for myocardial infarction.^56^ Hydrogel-based models of whole tissues could instead study the consequences on hemodynamics after the implantation of a tissue-engineered construct in the circulation, thereby providing information on the flow patterns and potential damages at the surrounding tissues. Despite the promising potential of computational models, several limitations still need to be overcome before their broader clinical application. For example, proper input data is required for the correct function of the models. This includes high-resolution patient scans and detailed 3D models of the patient’s tissue anatomy, which are not yet routinely available. In this context, a balance between the incorporated level of details and the computational costs should be found to achieve functional outcomes within a reasonable amount of time. Still, it can be assumed that, if used as diagnostic tools, models could improve the clinical efficiency of cell-based treatments by providing a safe localization of the injection site, a clear understanding of the mechanism behind tissue remodeling, and a patient-specific solution to ensure long-term clinical outcomes. In this context, computational models could foresee the effect of the patient’s specific anatomy on the success rate of the intervention, helping a tailored pre-operative planning and post-operative assessment of the surgical intervention.^57-59^ To conclude, besides focusing on the development of computational models regarding the specific behavior of (healthy) ECM-producing cells, it is also important to model how cell-mediated growth and remodeling interact with other regenerative processes as a function of patient-specific conditions. Ultimately, such models studying complex cellular interactions could easily be adapted to stem cell-based biomimetic TE therapies, often by relatively straightforward adjustments to the parameter space.

## Additive Manufacturing Technologies for Cardiovascular Tissue Engineering

The Food and Drug Administration has recently acknowledged the advancement of additive manufacturing (AM) technologies in the medical field and has released new guidelines on the use of 3D bioprinting for the development of medical devices. In this context, AM applications are subdivided into 4 categories: organ models, permanent non-bioactive implants, local bioactive and biodegradable implants, and whole tissues and organs.^60^ In the cardiovascular field, such emerging technologies are widely used to recreate regenerative 3D models of patient-specific anatomy to improve diagnostics and treatment.^61^ In addition to restoring the function of diseased tissues, 3D bioprinted substitutes may reduce triggering the immune response through the use of autologous or hiPSC-derived cells. Importantly, such tools are crucial for the development of personalized vascular stents to manage blood flow obstructions, the generation of biodegradable scaffolds to enable regeneration following a stroke, or the incorporation of primary cells or hiPSCs into 3D biomaterials to treat structural heart diseases. These methods utilize biomaterials such as alginate, collagen, gelatin, and decellularized ECM, which allows freedom in the combination of substrate and bioprinter to create the optimal environment for cell adhesion and proliferation for each specific tissue type.^62^ To be mentioned, Lee et al. developed 3D printed crosslinked gelatin hydrogels combined with hiPSC-derived cardiomyocytes, which resulted in tissues with organized sarcomeres and increased contractile forces.^63^ During the last decade, AM has also been used to fabricate customized heart valve prostheses to further enhance the patient’s anatomy accuracy and decrease host immune rejection, consequently improving the success rate of heart valve replacements. In 2012, Hockaday et al. built aortic valve hydrogels of poly-ethylene glycol-diacrylate (PEG-DA) and alginate by means of 3D printing/photo-crosslinking and seeding with porcine aortic valve interstitial cells, thereby paving the way for using AM in heart valve fabrication.^64^ This study showed the principal feasibility and advantages of using AM as fabrication tool for complex 3D geometries, such as heart valves, as well as the ability to tailor polymer stiffness during the bioprinting process. Nonetheless, improvements on the structural mechanical properties still need to be performed to guarantee the safe long-term performance of the 3D printed constructs. On the other hand, Coulter et al. have also demonstrated the possibility of using AM for the digital fabrication of tissues similar to aortic heart valves, featuring customizable geometry and leaflet architectures that resemble those of native tissues.^65^ In this study, heart valve and stent were fabricated simultaneously by combining spray and extrusion-based AM processes and biocompatible silicones. In vitro cyclic testing of printed prosthesis showed excellent hemodynamic performance under physiological pressure conditions.

Recently, advanced AM technologies such as melt electrowriting (MEW) and focused rotary jet spinning (FRJS) were able to further improve the mechanical properties of 3D printed prostheses, by providing highly structured and organized fibrous scaffolds^18,66^ (Fig. 1C, 1D). MEW is an emerging technology exploiting the principles of electrospinning and AM, thereby enabling the fabrication of solvent-free biodegradable scaffolds with micrometric features.^67^ Medical-grade PCL was used by N.T. Saidy to produce tunable tensile J-shaped stress/strain curves, anisotropy, and viscoelastic properties typical of native heart valve leaflets.^17,68^ Based on these results, they further implemented a new MEW platform that allows the user to manufacture highly tunable, spatially heterogeneous, fibrous tubular scaffolds with controlled mechanical properties containing elastin-like hydrogels for tailored microporosity and cellular infiltration. FRJS is an AM method that uses centrifugal jet spinning to rapidly form polymeric micro- and nanofibers, which are then focused and spatially patterned by means of a controlled airstream. FRJS is a platform able to rapidly manufacture helically and circumferentially aligned tissue-engineered constructs with preserved biomechanics.^18^ This technology holds a great promise for future structural heart valve replacement, as well as myocardial tissue repair, and many additional applications.

Different from the reconstruction of prostheses for structural heart diseases, AM is also used in stent fabrication for a wide range of applications, such as ureteral, oesophageal, pancreatic, airway, and vascular. Stent devices for transcatheter vascular or valvular replacement are crucial elements that enable prosthesis delivery and provide instant support after deployment. Stented heart valve replacements, for example, can be designed starting by measuring the size of the internal valve annulus diameter by either a computed tomography (CT) scan, echocardiography, or balloon sizing. Despite the technological advancement of such tools, size mismatch between the stent and the valvular annulus cannot be completely excluded, resulting in functional impairment and/or paravalvular leakage. In the context of patient-specific therapies, the particular aortic root anatomy of each single patient could be modeled allowing the customization of stents and prostheses. Here, computational modeling strategies combined with fused deposition modeling technology helped with the realization of a first polymeric 3D printed stent prototype for transcatheter heart valve applications.^69^

## Technologies That Have Reached Clinical Evaluation

**Figure 2.**
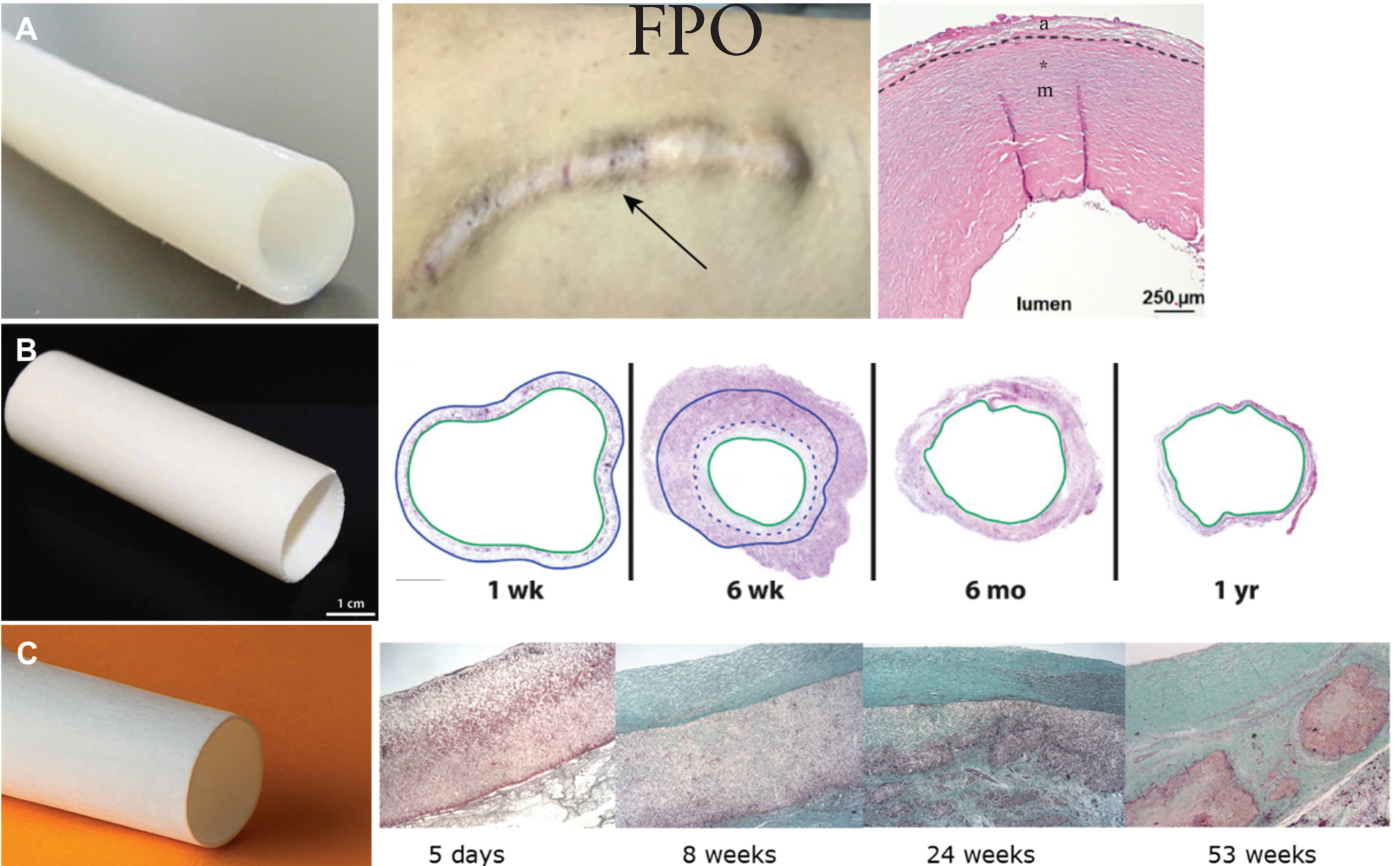
Examples of tissue engineered prostheses that have advanced into clinical trials. **A**: Representative figures of the successful outcome of the human acellular vessels developed by Humacyte for dialysis access.^41,70,71^ The figures depict a human acelluar vessel before implantation, when implanted beneath the skin after 6 years, and a histological section at 122 weeks. **B**: A tissue-engineered vascular graft pre-seeded with autologous bone marrow-derived mononuclear cells onto biodegradable scaffolds for single ventricle palliation developed by Shinoka and Breuer.^42^ The histological stainings of the pre-seeded vascular graft confirmed the in-vivo stenosis reversal after 1-year follow-up. **C**: Representative images of the Xeltis vascular graft used as extracardiac cavopulmonary conduit.^43^ Histology indicating the stages of cellularization and remodeling up to 53 weeks post-implantation. Figures were adapted and reprinted under Creative Commons CC-BY License and with permissions from the journals.

The successful combination of the above-mentioned tools and technologies has been demonstrated with encouraging outcomes in several clinical studies addressing structural cardiovascular defects (Fig. 2). Surgical intervention for heart or vascular diseases is usually performed with artificial, autologous, allogenic, or xenogeneic substitutes. Despite the relatively good performance of such options, none of them offer growth and regenerative capacities, which remains an important implication for pediatric cardiac surgery. The urgency to find a material with sufficient growth potential and durability to accommodate children’s needs and eliminate further invasive surgeries led to the pioneering implementation of tissue engineering concepts into clinical trials. In the past 6 years, clinical trials using ECM-based vascular grafts have shown promising results in terms of graft patency, regeneration, and host cell recellularization (NCT01744418, Table 2, Fig. 2A).^41,70,71^ Synthetic bioresorbable polymers either combined with one-step pre-seeding procedures^42^ or completely acellular grafts^43^ have also been advanced into clinics (Table 2). Pre-seeding procedures of autologous bone marrow-derived mononuclear cells onto biodegradable scaffolds have been applied to generate tissue-engineered vascular grafts for single ventricle palliation (Fig. 2B). The clinical application of such grafts in Fontan patients has shown successful evaluation for safety and efficacy, as well as host cell repopulation and regeneration over time. Despite the observation of stenotic events after 8-month follow-up in the U.S. clinical trial (IDE 14127), the integration of in silico computational modeling tools into the in vivo adaptation of these grafts allowed the prediction of a spontaneous resolution via inflammation-driven graft remodeling.^42^ This evidence becomes of major importance for future clinical studies, which should implement computational modeling tools to reduce trial-and-error validation procedures.

Furthermore, bisurea-based and ureido-pyrimidone-based supramolecular polymers are under investigation in early clinical trials (Table 2, Fig. 2C). Congenital heart disease, such as univentricular cardiac malformation have been treated via extracardiac cavopulmonary bypass without observing device-related adverse events and reporting sustained patency and performance at 2 years^72^ (NCT02377674). Mixed outcomes have been however reported for the Xplore-1 and Xplore-2 clinical trials (NCT02700100 and NCT03022708), therefore requiring further investigations with longer follow-up periods to determine the efficacy of polymer-based valved conduits.^34^ In fact, preclinical studies investigating the regenerative properties of such supramolecular materials have reported slow polymeric degradation rate as well as heterogeneous remodeling with regard to cell infiltration, scaffold resorption, and ECM deposition within the same valve explant.^73,74^

## Conclusion

Tissue-engineered constructs offer a great potential to meet the increasing needs of patients in many cardiovascular fields, and particularly to accommodate the growth requirements of pediatric patients. For decades the gold standard of care has been autologous grafts replacements with understandable drawbacks such as limited supply and regeneration. Xenogeneic alternatives are often preferred choices to synthetic prostheses, however, functional failures, inflammation, and maladaptive remodeling are frequently observed, limiting the quality of the patient’s life. Pre-seeded and/or decellularized tissue-engineered replacements may overcome such clinical shortcomings, by providing biomimetic substitutes that can completely integrate within the host and the native tissues, grow, and remodel over time. However, several challenges need to be mastered before broad clinical translation will be possible. Next generation technologies and tools (eg, cell technologies, gene editing, computational modeling, and additive manufacturing) may support this translation by establishing simplified and scaled-up manufacturing processes to fasten and increase the offer to patients. Strong preclinical and recent first clinical successes have demonstrated the huge clinical potential of such tissue engineering technologies, however, further research and development are needed to accelerate their adoption into clinical routine.

## Data Availability

No new data were generated or analyzed in support of this research.
